# Supplementary material for: Evidence-Based Interventions for Reducing Breast Cancer Disparities: What Works and Where the Gaps Are?
Source: Cancers (Basel). 2022 Aug 26;14(17):4122. doi: 10.3390/cancers14174122 (PMC9455068; doi:10.3390/cancers14174122)
Supplement: Supplementary file 1 [file cancers-14-04122-s001.zip › cancers-1843442-supplementary.pdf]

Supplementary Materials

Table S1. Summary of the EBCCP scoring procedures

<table>
<tr><td colspan="3">EBCCP scores (1.0/low to 5.0/high): This site provides a consumer-reports-like list of programs that have been reviewed by a panel of topic experts in the field. Programs are rated on 3 criteria which include the following: research integrity, intervention impact, and dissemination capability. For more detailed information, please refer to the program ratings.</td></tr>
<tr><th>Category</th><th>Definition</th><th>Scoring</th></tr>
<tr><td>Research integrity</td><td>Integrity reflects the overall confidence reviewers can place in the findings of a program's evaluation based on its scientific rigor. The research integrity rating system comprises 16 criteria scored by external peer reviewers. Scores on each criterion range, on a 5-point scale, from low quality to high quality. The overall integrity score is a weighted average of the 16 criteria reflecting the merits of the science that went into the program evaluation.</td><td>5 - high confidence in results, findings fully defensible
4 - strong, fairly good confidence in results
3 - mixed, some weak, some strong characteristics
2 - weak, at best some confidence in results
1 - little or no confidence in results

The 16 scored criteria include:
1. Theory-/hypothesis driven measure selection
2. Reliability
3. Validity
4. Intervention fidelity
5. Nature of comparison condition
6. Comparison fidelity
7. Assurances to participants
8. Participant expectations
9. Standardized data collection
10. Data collection bias
11. Selection bias
12. Attrition
13. Missing data (other than missing data resulting from attrition)
14. Analysis meets data assumptions
15. Hypothesis-driven selection of analytic methods
16. Anomalous findings
(For definitions of each criterion, refer to: https://ebccp.cancercontrol.cancer.gov/ratingsDetail.do)</td></tr>
<tr><td>Intervention impact</td><td>Intervention impact is calculated based on population reach and intervention effect size that are rated separately and combined into a single score based on the effect size score and combined intervention impact score à</td><td><table>
<tr><th>Reach Score</th><th>Effect Size Score</th><th>Combined Intervention Impact Score</th></tr>
<tr><td rowspan="3">1 = Low Reach – The study or studies excluded or probably excluded a high proportion of members of the defined target population (i.e., defined according to demographic and/or risk factor characteristics). The intervention tested was not representative of the target population.</td><td>1 – Small</td><td>1</td></tr>
<tr><td>3 – Medium</td><td>2</td></tr>
<tr><td>5 – Large</td><td>3</td></tr>
<tr><td rowspan="3">3 = Moderate Reach – The study or studies excluded or probably excluded a small but significant proportion of members of the defined target population. The intervention tested may be only partially representative of the target population.</td><td>1 – Small</td><td>2</td></tr>
<tr><td>3 – Medium</td><td>3</td></tr>
<tr><td>5 – Large</td><td>4</td></tr>
<tr><td rowspan="3">5 = Broad Reach – The study or studies included virtually all relevant members of the defined target population. The intervention tested was representative of the target population.</td><td>1 – Small</td><td>3</td></tr>
<tr><td>3 – Medium</td><td>4</td></tr>
<tr><td>5 – Large</td><td>5</td></tr>
</table></td></tr>
</table>

**Dissemination capability**	The readiness of program materials for use by others as well program's capability to offer services/resources to facilitate dissemination. This is measured through (a) the quality of implementation materials; (b) training and technical assistance protocols; and (c) the availability of quality assurance materials to determine whether their implementation is done with high fidelity to the original model.	5 - high quality materials with technical assistance readily available and training/qualified trainers readily available OR technical assistance/training not needed/not necessary<br>4 - high quality materials, limited technical assistance and/or training/qualified trainers available<br>3 - materials of sufficient quality with limited technical assistance and/or training/qualified trainers available<br>2 - materials available, but of low quality or very limited in scope; training/qualified trainers and technical assistance either not available or limited<br>1 - materials, training and technical assistance not available; in case of model that requires no curriculum (i.e., therapeutic models), training/qualified trainers and technical assistance not available

RE-AIM scores (% or N/A): The goal of RE-AIM is to encourage program planners, evaluators, readers of journal articles, funders, and policy-makers to pay more attention to essential program elements including external validity that can improve the sustainable adoption and implementation of effective, generalizable, evidence-based interventions.

The five steps to translate research into action are: 1) Reach the target population, 2) Effectiveness or efficacy, 3) Adoption by target settings or institutions
Implementation (consistency of delivery of intervention), 4) Maintenance* of intervention effects in individuals and settings over time.
*Please note that "Maintenance" is not calculated as part of the RE-AIM scoring process for EBCCP.

**Category**	**Definition**	
**Reach**	The absolute number, proportion and representativeness of individuals who are willing to participate in a given initiative, intervention or program	
**Effectiveness**	The impact of an intervention on important effects, quality of life and economic outcomes.	
**Adoption**	The absolute number, proportion and representativeness of settings and intervention agents (people who deliver the program) who are willing to initiate a program.	
**Implementation**	At the setting level, implementation refers to the intervention’s fidelity to the various elements of an intervention’s protocol, including consistency of delivery as intended and the time and cost of the intervention. At the individual level, implementation refers to client’s use of the intervention strategies.	
